# Effect of hepatitis B vaccination on HBV-infection among school children in Yaounde; ten years after the introduction of HBV vaccine into routine Immunization Program in Cameroon

**DOI:** 10.11604/pamj.2024.47.169.40369

**Published:** 2024-04-05

**Authors:** Philippe Salomon Nguwoh, Christian Taheu Ngounouh, René Ghislain Essomba, Patrice Zanga Olinga, Julienne Louise Ngo Likeng, Gilbert Nguepidjo, Sandrine Chimène Tonmeu Douyong, Désiré Tchoffo, Anne Esther Njom Nlend, Marie Claire Okomo Assoumou, Joseph Fokam

**Affiliations:** 1 National Public Health Laboratory, Ministry of Public Health, Yaounde, Cameroon,; 2 Distant Production House University, Delaware, United State of America,; 3 Higher Institute of Health Professions, Faculty of Medicine and Pharmaceutical Sciences, University of Douala, Yaounde, Cameroon,; 4 Higher Institute of Sciences and Techniques Applied to Health, Faculty of Medicine and Pharmaceutical Sciences, University of Douala, Yaounde, Cameroon,; 5 Faculty of Health Sciences, University of Lisala, Lisala, Mongala, Democratic Republic of Congo (DRC),; 6 Faculty of Medicine and Biomedical Sciences, University of Yaounde 1, Yaounde, Cameroon,; 7 School of Health Sciences, Catholic University of Central Africa, Yaounde, Cameroon,; 8 Higher Institute of Medical Technology, Faculty of Medicine and Pharmaceutical Sciences, University of Douala, Yaounde, Cameroon,; 9 Faculty of Health Sciences, University of Buea, Buea, Cameroon,; 10 Chantal BIYA International Reference Centre for Research on HIV/AIDS prevention and management, Yaounde, Cameroon

**Keywords:** Effect, HBV-infection, vaccination status, children, Yaounde

## Abstract

**Introduction:**

since the introduction of the anti-HBV vaccine into the Expanded Program on Immunization (EPI) in 2005 in Cameroon, vaccination coverage has reached 99.0%. This coverage would indicate an increase in the number of children immune to Hepatitis B Virus (HBV) and a decrease in susceptibility to HBV-infection. This study was conducted to evaluate the effect of the HBV vaccine on pediatric HBV-infection in Yaounde, Cameroon.

**Methods:**

this school-based cross-sectional study was conducted from February to May 2016 among 180 children from Nkomo public school. The study population was stratified into two groups: vaccinated (n=95) versus (vs) unvaccinated (n=85). Screening for HBV biomarkers was done using a rapid panel test for detection (HBsAg, HBeAg and anti-HBc) and anti-HBs titer using enzyme linked immunosorbent assay (ELISA). Statistical analyses were done using SPSS v. 22 with p < 0.05 considered significant.

**Results:**

the mean age was 9.65 years. HBsAg (p=0.019) and anti-HBc (p=0.001) rates were detected in children aged ≥10 years and children aged < 10 years (95.95% [71/74]) were vaccinated vs 22.64% (24/106) for those aged ≥10 years (OR: 80.86; 95% CI: 23.36%-279.87%, p < 0.0001). According to anti-HBV vaccination status, HBsAg rate varied from [9.41% (8/85) to 1.05% (1/95), p=0.025], HBeAg rate varied from [2.35% (2/85) to 0% (0/95), p= 0.42] and anti-HBc rate ranged from [12.94% (11/85) to 2.10% (2/95), p= 0.011].

**Conclusion:**

despite the variability of the anti-HBs titer, vaccination against HBV has a positive effect on the reduction of HBV-infection in children in tropical settings such as Cameroon.

## Introduction

HBV-infection remains a major public health problem worldwide, with about 248 million people chronically infected and more than 686.000 deaths per year as a result of HBV-related liver failure and hepatocellular carcinoma (HCC) [1]. Low and middle-income countries of Asia, Western pacific and sub-Saharan Africa (SSA) bear the brunt of the disease with high prevalence (HBsAg>8%) of their populations being hepatitis B surface antigen (HBsAg) positive [2]. In these areas of high prevalence of hepatitis B, the most common routes of HBV-infection are mother-to-child transmission (MTCT) at birth and horizontal transmission from close contacts during early childhood [3,4]. Of note, the routes of HBV transmission encountered worldwide in children are horizontal transmission (contacts with infected family members or objects contaminated with blood), frequently observed in SSA and vertical or perinatal transmission (from mother-to-child during childbirth or breastfeeding) encountered in Asia [5]. Therefore, the risk of transmission of the virus is higher in children born to mothers positive for both HBsAg and HB-e- antigen (HBeAg) of the HBV (70 to 100% in Asia and 40% in Africa) than those born to HBsAg positive mothers only (5-30% in Asia and 5% in Africa) with a viral load > 200,000 IU/ml [6-8]. Subsequently, HBV-infection at an early age is associated with risks of chronic infection (90%) and HCC (up to 50%), children in high endemic settings, such as sub-Saharan Africa (SSA), deserve special attention [9,10]. These factors associated with chronic carriage status include resident status, history of blood transfusion, and history of surgery, previous HBsAg tests, and lack of vaccination history [11]. However, an active immunization with hepatitis B vaccine among all pregnant with HBsAg negative after screening of HBV, providing HBV antibodies at birth to all vertically-exposed babies, and a universal vaccination of newborns remains the effective measure to prevent and control HBV-infection [12,13]. We also note that an antiviral therapy in the last stages of pregnancy is the most effective way to reduce mother-to-child transmission (MTCT) of HBV-infection [14,15]. As recommended by the World Health Organization (WHO) in 1992, practically all countries globally have introduced hepatitis B vaccine into national infant immunization schedules to prevent chronic HBV-infection and the associated disease burden [16]. Nevertheless, of country specific prevalence, WHO has recommended at least three doses of hepatitis B vaccine for all babies, including a first dose within 24 hours of birth [17]. Cameroon is considered as a high endemicity country of hepatitis B (HBsAg>8%). The prevalence of HBsAg positivity among pregnant women attending the antenatal care (ANC) is estimated at >10% [18,19], suggesting systematic large-scale universal screening for hepatitis B in all women of childbearing age and routine vaccination for those with negative HBsAg results. Meanwhile, little is known about the prevalence of HBsAg among children born to infected mothers. The vaccination against hepatitis B was introduced into the EPI in Cameroon in 2005, using the WHO-recommended schedule at 6, 10, and 14 weeks of age, with an estimated coverage of 99.0% [20,21]. Data on the effect of vaccination on the prevalence of HBV-infection in pediatric settings since the introduction of routine vaccination in newborns are somewhat scarce. Several studies conducted in SSA have reported a prevalence of HBsAg of 3.0% and 2.0% among children vaccinated vs 6.2% and 11.8% of unvaccinated children [12,22]. Moreover, the study conducted in Cameroon in 1991 before the introduction of HBV vaccine into the EPI has reported a high rate of HBsAg (19.9%) among school children [23]. All these concordant studies on the epidemiological importance of HBV-infection underscore the need for routine vaccination in SSA countries particularly in Cameroon and the establishment of a monitoring of the post-immunization response to ensure protection against HBV-infection in tropical settings such as Cameroon. The general objective of this present study was to evaluate the effect of hepatitis B vaccination on HBV-infection ten years after the introduction of hepatitis B vaccine into routine EPI in Cameroon. The specific objectives were: (i) to determine HBV biomarkers prevalence according to characteristics of the study population, (ii) to categorize the post-immunization response according to the characteristics of the study population, and (iii) to compare the post-immunization response according to HBV biomarkers.

## Methods

**Study design and setting**: this study was designed to evaluate the effect of hepatitis B vaccination on HBV-infection among school children in Yaounde ten years after its introduction of the HBV vaccine into routine EPI in Cameroon. This school-based cross-sectional study was conducted from February 8^th^ to May 18^th^, 2016 among 180 nursery and primary school children at Nkomo, Yaounde. The Nkomo public school is located in the Yaounde IV district opposite the Nkomo district medical center and are made up of several groups with estimates of at least more than 1000 pupils.

**Participants**: a non-probabilistic method was used to select school children. So, was included in this study, nursery and primary school children uninfected by HBV, aged between 3 to 14 years old, at Nkomo. The participants were stratified in two groups [panel A: vaccinated with the complete three doses of the HBV vaccine (Zilbrix Hepta: 10μg HBsAg) vs panel B: unvaccinated]. Excluded from this study were school children with incomplete data and from whom blood samples could not be obtained.

**Data and specimen collection**: the school children were selected consecutively during the study period and the general characteristics data such as age, sex, vaccination status have been collected using a standard questionnaire. The selection of vaccinated children was done under presentation of the vaccination card with the complete three doses of the HBV vaccine (pentavalent vaccine: DTC-HepB-Hib1) received according to the schedule in force for the age group concerned. From each school children enrolled, 4 to 5 ml of whole blood samples were collected aseptically by veno-puncture in the dry vacutainer tubes and the sera obtained after centrifugation was spared into Eppendorf tubes and stored at -20°C until laboratory analysis.

**Variables**: both quantitative and qualitative variables were collected in this current study. The quantitative variables collected were age and anti-HBs antibody titer for the post-immunization response. Post-immunization response was interpreted as negative, positive and protective response with anti-HBs antibody titer: <1.0 IU/L; ≥1.0<10.0 IU/L and ≥ 10.0 IU/L respectively. The qualitative variables collected were sex, vaccination status and the interpretation (positive vs negative) of HBV biomarkers (HBsAg, HBeAg and anti-HBc).

**Data sources/measurement of variables**: the data were collected among nursery and primary school children at Nkomo, Yaounde. The quantitative variables such as age and the qualitative variables such as sex, vaccination status have been collected in the vaccination card of children using a standard questionnaire. Moreover, the quantitative variable such as anti-HBs antibody titer for the post-immunization response were measured using the ELISA. Furthermore, the qualitative variables such as HBsAg, HBeAg and anti-HBc were done using HBV rapid diagnostic test.

**Bias**: post-vaccination response was assessed at the time of study enrollment, with the possibility of loss of humoral response with increasing age. Performing the DNA molecular test would have elucidated any possibility of occult HBV. Moreover, the low sample size did not allow us to extrapolate results to the general population of children aged between 3 to 14 years old. This low sample size obtained could be explained by the lack of financial resources. Furthermore, lack data on nutritional status of school children were not available making the interpretation of our finding difficult, particularly in non-responders or negative response to the vaccine [12,13].

**Sample size determination**: the sample size was calculated using the following formula:


$$n=Z^{2}P\left(1-P\right)/d^{2}$$

=170 participants. With Z= the standard deviation of 1.96 (95% confidence interval); P= the prevalence of HBsAg positivity (19.9%) [23] among school children in Cameroon more than ten years before the introduction of HBV vaccine into EPI; d= random error (estimated at 6%).

**Quantitative variables**: in the current study, age of the school children and anti-HBs antibody titer for the post-immunization response were the only two quantitative variables recorded. Age was collected from the vaccination card and anti-HBs antibody titer was performed in the laboratory using ELISA.

**Laboratory testing of HBV biomarkers**: the samples were analyzed by the “OnSite HBV (HBsAg, anti-HBs, HBeAg, anti-HBe and anti-HBc) Rapid Panel Test” kit (from the CTK Biotech USA laboratory, Lot No F0624K7D00, date of manufacture: 10-08-2015 and expiration date: 09-02-2017) according to the manufacturer´s instructions.

**Detection of HBsAg**: the HBsAg biomarker was used to qualitatively detect any children infected with HBV, using lateral flow chromatographic immunoassay sandwich with 100µl of serum. Validated results were reported as HBsAg reactive (HBsAg positive) when two distinct red lines appeared (one line in the control zone “C” and another in the test zone “T”). Negative result shows the appearance of one red line only in control zone “C” and no appearance of red line in test zone “T” [24].

**Detection of HBeAg**: the HBeAg biomarker was used to qualitatively detect any children with HBV replication, using lateral flow chromatographic immunoassay sandwich with 100μl of serum. Validated results were reported as HBeAg reactive (HBeAg positive) when two distinct red lines appeared (one line in the control zone “C” and another in the test zone “T”). Negative result shows the appearance of one red line only in control zone “C” and no appearance of red line in test zone “T” [24].

**Detection of anti-HBc**: the anti-HBs biomarker was used to qualitatively detect any previous contact with HBV, using lateral flow chromatographic immunoassay competition with 100μl of serum. Validated results were reported as anti-HBc reactive (anti-HBc positive) when one red line appeared only in the control zone “C”. Negative result shows the appearance of two distinct red lines (one line in the control zone “C” and another in the test zone “T”) [24].

**Anti-HBs antibody titer**: the anti-HBs titration was done using ELISA, according to the manufacturer´s instructions (manufactured: CTK Biotech®, USA; batch: E0604K3C00) to assess the vaccination status against HBV-infection. The anti-HBs test is based on the principle of enzyme immunoassay according to the ELISA sandwich method. The absorbance measurement is taken within 15 minutes at 450 nm. The reference wavelength is between 630-690 nm. The different optical density (OD) obtained for the control sera must meet the following criteria: negative control: ≤0.10 and positive control: ≥0.80. The presence or absence of anti HBs antibody was determined by comparing the OD of the sample with the cutoff values. Therefore, according to the manufactured: anti-HBs titer <1.0 IU/L mean negative or no response, anti-HBs titer ≥ 1.0<10.0 IU/L mean positive or low response, and anti-HBs titer ≥10.0 IU/L mean good vaccine or protective response.

**Statistical analysis**: the collected data were included in the first Microsoft Excel version 2013 sheet, labelled and subsequently transported into the statistical data analysis IBM Statistical Package for Social Sciences (SPSS v. 22, Inc, Chicago). Descriptive analysis was done to reveal the absolute frequency (effective), relative frequency (percentage), modalities of the variables (quantitative and qualitative), the average age, the standard deviation, the median age and the percentiles. The prevalence of each HBV biomarker was calculated by dividing the effective of the modalities by the total number of the population multiplied by 100 and the results were expressed as a percentage. Means and/or medians were compared between the two groups using the Student's t test. The proportions of variables were compared using the chi-square test and the Fisher’s exact test. The study of associations was done by bivariate analysis and associations between the variables were sought with the Odds Ratio (OR), expressed with 95% confidence interval (CI). The probability was significant for all p-values less than 5% (p<0.05). The results obtained after analysis were presented in table form.

**Ethics statement**: in the current study, all methods were carried out in accordance with relevant guidelines and regulations. Before the study started, ethical approval was obtained (Approval No. 2016/07/789/CE/CNERSH/SP) from the National Ethics Committee of Research on Human Health of Cameroon. Also, Administrative authorization was issued by the departmental delegate of basic education of Mfoundi (Approval No. 397/L/MINEDUB/DREB-C/DDEB-MFDI/SSAPPS from February 2016). Written informed consent from parental or guardian or legal representative for children was obtained from each study participant prior to data collection and they participated voluntarily.

## Results

**Characteristics of the study population**: a total of 180 school children fulfilled the inclusion criteria was enrolled in this study. The mean age of the study population was 9.65±3.67, ranging from 3 to 14 years old. In panel-A, median and mean age were similar [median age: 8 (IQR: 5-12) years and mean age (±SD): 8.28 ±3.72 years]. Meanwhile, in panel-B, median age was 12 (IQR: 10-14) years with a mean age of 11.17±2.96 years. The distribution of the study population according to sex shows that, 52.22% (94/180) were girls and 47.78% (86/180) were boys (Table 1).

**Table 1:** characteristics of the study population

Group category characteristics	Panel A: vaccinated n=95 (%)	Panel B: unvaccinated n=85 (%)
Median age [IQR] years	8(5-12)	12(10-14)
Mean age ±STD years	8.28±3.72	11.17±2.96
Age (in years)		
< 10 (n=74)	71(95.95)	03(4.05)
≥ 10 (n=106)	24(22.64)	82(77.36)
Sex		
Girls (n=94)	52(55.32)	42 (44.68)
Boys (n=86)	43(50.00)	43(50.00)

The means were compared using the student’s t-test and the proportion using the Chi-X^2^ and Fischer’s test

**Distribution of HBV biomarkers according to the characteristics of the study population**: among 180 children tested, HBsAg rate was 5.0% (9/180), HBeAg detection was 1.11% (2/180) and 7.22% (13/180) was previously exposed to HBV (anti-HBc positive). Seroprevalence of HBsAg (p=0.019) and anti-HBc were only detected in children aged ≥10 years (p=0.0014). Moreover, the current study shows that, HBsAg (6.98% vs 3.19%) and anti-HBc (9.30% vs 5.32%) rates were more detected in boys than girls. Meanwhile, the similar results were observed between boys and girls for the HBeAg positivity. According to anti-HBV vaccination status, 52.78% (95%CI: 45.21-60.25; 95/180) of children were vaccinated vs 47.22% (95%CI: 39.75-54.79; 85/180) for those unvaccinated. HBsAg positivity rates varied from [9.41% (8/85) to 1.05% (1/95), p=0.025], HBeAg positivity rates varied from [2.35% (2/85) to 0% (0/95), p=0.42] and anti-HBc detection rates were [12.94% (11/85) to 2.10% (2/95), p=0.011] (Table 2).

**Table 2:** distribution of HBV biomarkers according to the characteristics of the study population

	Serological HBV biomarkers					
Characteristics	HBsAg+ n= 09 (%)	p-value	HBeAg+ n= 02 (%)	p-value	anti-HBc+ n=13 (%)	p-value
**Age (in years)**						
**< 10 (n=74)**	0(0.00)	0.019**	0(0.00)	0.42	0(0.00)	0.0014**
**≥ 10 (n=106)**	09(100.0)		02(100.0)		13 (100.0)	
**Sex**						
**Girls (n=94)**	03(3.19)	0.4	01(50.00)	1.0	05(5.32)	0.30
**Boys (n=86)**	06(6.98)		01(50.00)		08(9.30)	
**Pupils anti-HBV vaccination status**						
**Vaccinated (n=95)**	01(1.05)	0.025**	0 (0.00)	0.42	02(2.10)	0.011**
**Unvaccinated (n=85)**	08(9.41)		02(2.35)		11(12.94)	

** significant value

**Distribution of post-immunization response according to characteristics of the study population**: among vaccinated children, 82.11% (78/95) developed a negative or no response (anti-HBs titer <1.0 IU/L), 13.68% (13/95) developed a positive or low response (anti-HBs ≥1.0<10.0 IU/L) and only 4.21% (4/95) developed a good or protective immune response (anti-HBs titer ≥10.0 IU/L) against HBV-infection. Seventy-four point sixty-five percent (74.65% (53/71)) vs 83.33% (20/24) respectively (children aged: <10 vs≥10 years old) had a negative post-immunization response and this negative response was more observed in girls. Ten children (14.08%) aged < 10 years vs 12.50% (3/24) for those aged ≥10 years had a positive post-immunization response and this positive response was more detected in boys. The children aged <10 years developed a protective immune response than those aged ≥ 10 years (4.22% [3/71] vs 4.17% [1/24]) and this protective response was more observed in boys. The difference observed was not statistically significant (p<0.05) (Table 3).

**Table 3:** distribution of post-immunization response according to characteristics of the study population

Characteristics	No or Negative response anti-HBs <1.0 IU/L	p-value	Low or Positive response anti-HBs≥ 1.0<10.0 IU/L	p-value	Protective response anti-HBs ≥ 10.0 IU/L	p-value
**Age (in years)**						
**< 10 (n=71), n (%)**	53(74.65%)	0.5	10(14.08%)	0.8	03(4.22%)	0.5
**≥ 10 (n=24), n (%)**	20 (83.33%)		03 (12.50%)		01(4.17%)	
**Sex**						
**Girls (n=52), n (%)**	41 (78.85%)	0.6	06(11.53%)	0.5	02 (3.85%)	0.7
**Boys (n=43), n (%)**	32(74.42%)		07(16.28%)		02(4.65%)	

**Distribution of post-vaccination response according to HBV biomarkers**: this distribution shows that, HBV biomarkers (HBsAg [1.28%] and anti-HBc [5.13%]) were detected in children with negative response (anti HBs <1.0 IU/L). On the other hand, no HBV biomarkers were found in children with positive response (anti HBs ≥1.0≥10.0 IU/L) and protective response (anti HBs ≥10.0 IU/L) (Table 4).

**Table 4:** distribution of post-immunization response according to HBV biomarkers

	Serological HBV biomarkers					
Post-immunization response	HBsAg+ n= 09 (%)	p-value	HBeAg+ n= 02 (%)	p-value	anti-HBc+ n=13 (%)	p-value
**Negative response anti HBs <1.0 IU/L**	01 (1.28)	0.09	0(0.00)	0.6	04(5.13)	0.3
**Positive response anti HBs≥ 1.0<10.0 IU/L**	0 (0.00)	0.8	0(0.00)	0.3	0(0.00)	0.5
**Protective response anti HBs ≥ 10.0 IU/L**	0(0.00)	0.4	0(0.00)	0.02**	0 (0.00)	0.8

** Significant value

## Discussion

This study was designed to evaluate the effect of hepatitis B vaccination on HBV-infection ten years after the introduction of hepatitis B vaccine into routine EPI in Cameroon by comparing HBV biomarkers seroprevalence among vaccinated and unvaccinated children. In the current study, the overall HBsAg positivity was 5.0% (95% CI: 2.31-9.28; 9/180), showing a dramatic decrease as compared to the high HBsAg prevalence (19.9%) among school children reported in 1991 by [23], more than ten years before the introduction of the HBV vaccine into EPI in Cameroon. Similar findings were found in the Republic of Senegal [25,26] where it was reported a significantly decreased HBsAg seroprevalence among children. In fact, we expected this low seroprevalence of 5.0% despite the fact that Cameroon is located in an area of high endemicity for the HBV-infection (HBsAg >8.0%) [27], and the mean age (9.65 ± 3.67 years old) of study population, meaning that the majority of them benefited from the routine EPI vaccine in 2005. This result indicates that the interventions implemented in the past ten years since the introduction in 2005 of the HBV vaccine into the EPI in Cameroon have had a significant effect on reducing HBV-infection. These findings corroborate with several studies worldwide concerning the importance of the HBV vaccine in newborns [12,28,29,30,31]. Our findings revealed that, the positivity rate of HBsAg (p=0.019) and anti-HBc (p=0.0014) was observed significantly in children, aged ≥10 years meaning that the HBV- infection increased with age. Despite the low sample size, this high seroprevalence of HBV biomarkers among children aged ≥10 years supports the role of MTCT and early high horizontal transmission [12]. In the same vein, the rates of HBsAg (p=0.025) and anti-HBc (p=0.011) were significantly lower among children who have been vaccinated than unvaccinated children respectively.

These findings corroborate with those of many surveys that have reported a decreased HBsAg prevalence among children who have been vaccinated than those unvaccinated [12,22]. Also, several studies conducted an African country, Asia and Russia which have shown the prevalence of HBsAg to have declined significantly since the introduction of the hepatitis B vaccination programs [21,29,30,32,33]. In view of these results, it is a priori established, that vaccination reduced the carriage rate of HBsAg 9 times and that of anti-HBc 6 times, indicating that, depending on the duration of the vaccination program, the number of people immunized against HBV-infection will increase and as a result they will be less likely to contract the disease and the rate of transmission will drop significantly. Regarding the post-immunization response, it appears that the majority of children (>50%) had a negative or no post-immunization response (anti HBs titer <1.0 IU/L) and this negative response was more observed in girls than boys. This low level of immunity in fully vaccinated children or non-responder children may be explained by the fact that the quality of vaccine may be compromised by storage conditions (broken cold chain, supply chain, health staff…), age category, socioeconomic conditions and immune system [12,13,21,34]. This result can be also explained by the nutritional status of children which could have an impact on antibody response to vaccination [12]. Additionally, the HBV mother status could be another factor explaining the low immunity because we found that 60.0% were HBV positive. Furthermore, ten children (14.08%) aged <10 years vs 12.50% (3/24) for those aged ≥10 years had a positive post-immunization response and this positive response was more detected in boys. The children aged <10 years developed a protective immune response than those aged ≥10 years (4.22% [3/71] vs 4.17% [1/24]) and this protective response was more observed in boys. In contrast with several studies conducted worldwide, our findings are lower than those observed by Lô G *et al*. and Greengold B *et al*. [30,34] which have reported a better protective response among vaccinated children. This difference could be explained by the duration of the evaluation of the post-vaccination response [33].

Also, the age of the participants which amounts to say that the level of anti-HBs decreases as age increases [29]. Although our results are weak, it adds to the various studies conducted in Cameroon [13,21]. This highlights the need for strengthening the immunization programs with the administration of additional HBV vaccine booster doses [30]. Concerning the post-immunization response according to HBV biomarkers, the present study found that HBsAg and anti-HBc were detected in children with negative or no response. Meanwhile, no HBV biomarkers were found in children with positive or low response and protective response. Despite the small number of positive and protective responses observed in this study, the successful introduction of HBV vaccine into the EPI to fight against HBV-infection in Cameroon had a positive impact on the HBV prevalence among vaccinated children with a positive response. Thus, our findings confirm that vaccination of infants contributes to the reduction of the HBV biomarkers prevalence.

## Conclusion

This study aimed to evaluate the effect of the introduction of hepatitis B vaccine in Cameroon by comparing HBV biomarkers between vaccinated vs unvaccinated children and to estimate the immunization of the HBV vaccine. The study reveals a dramatic decrease as compared to the high HBsAg prevalence among school children reported in 1991, more than ten years before the introduction of the HBV vaccine into EPI in Cameroon. Furthermore, the rate of HBsAg and anti-HBc were significantly lower among children who have been vaccinated than unvaccinated children. Despite the low level of immunity in fully vaccinated school children and the low rate of protection, this study indicates that the interventions implemented in the past 10 years have had a significant great effect on reducing the risk of HBV-infection.

### What is known about this topic

- *HBV-infection is a major public health problem worldwide with about 248 million people chronically infected and more than 686,000 deaths each year as a result of HBV-related liver failure and hepatocellular carcinoma (HCC);*
- *Low and middle-income countries of Asia, Western pacific and sub-Saharan Africa (SSA) such as Cameroon bear the brunt of the disease with high prevalence (HBsAg>8%);*
- *The vaccination against hepatitis B was introduced into the EPI in Cameroon in 2005, using the WHO-recommended schedule at 6, 10, and 14 weeks of age, with an estimated coverage of 99.0%*.

### What this study adds

- *The seroprevalence of HBV biomarkers using HBV rapid panel test between vaccinated vs. unvaccinated children;*
- *Significantly lower seroprevalence of HBsAg and anti-HBc among children who have been vaccinated than unvaccinated children; so, vaccination reduced the carriage rate of HBsAg 9 times and that of anti-HBc 6 times;*
- *Depending on the post-immunization response, this study showed that although the children were vaccinated, almost 76.84% (73/95) did not respond to vaccination*.
